# *In vivo* single-molecule kinetics of activation and subsequent activity of the arabinose promoter

**DOI:** 10.1093/nar/gkt350

**Published:** 2013-05-03

**Authors:** Jarno Mäkelä, Meenakshisundaram Kandhavelu, Samuel M. D. Oliveira, Jerome G. Chandraseelan, Jason Lloyd-Price, Juha Peltonen, Olli Yli-Harja, Andre S. Ribeiro

**Affiliations:** ^1^Laboratory of Biosystem Dynamics, Computational Systems Biology Research Group, Department of Signal Processing, Tampere University of Technology, FI-33101 Tampere, Finland and ^2^Institute for Systems Biology, 1441N 34th Street, Seattle, WA 98103-8904, USA

## Abstract

Using a single-RNA detection technique in live *Escherichia coli* cells, we measure, for each cell, the waiting time for the production of the first RNA under the control of P*_BAD_* promoter after induction by arabinose, and subsequent intervals between transcription events. We find that the kinetics of the arabinose intake system affect mean and diversity in RNA numbers, long after induction. We observed the same effect on P*_lac/ara-__1_* promoter, which is inducible by arabinose or by IPTG. Importantly, the distribution of waiting times of P*_lac/ara-__1_* is indistinguishable from that of P*_BAD_*, if and only if induced by arabinose alone. Finally, RNA production under the control of P*_BAD_* is found to be a sub-Poissonian process. We conclude that inducer-dependent waiting times affect mean and cell-to-cell diversity in RNA numbers long after induction, suggesting that intake mechanisms have non-negligible effects on the phenotypic diversity of cell populations in natural, fluctuating environments.

## INTRODUCTION

Transcription in *E. coli* is, at a genome-wide scale, a relatively rare stochastic event (1–3). Further, many genes only become active in response to external stimuli (4–7), via processes that are also stochastic (7). Although much is known on the noise in gene expression at the single-cell level (1–3,7–10), most of our present knowledge concerning the kinetics of response, in terms of gene activity, to external signals concerns the average behaviour of cell populations alone (11). However, to characterize the dynamics and the underlying steps of intake processes, it is necessary to observe their effects in individual live cells (12). This observation should inform also on the robustness of cellular response mechanisms by informing on the degree of change in the responses of a single cell to multiple occurrences of the same stimulus, as well as the difference in responses to different stimuli.

One of the most well-known gene activation mechanisms is the arabinose utilization system of *E. coli*. This system imports arabinose into the cell by AraFGH, an arabinose-specific high-affinity ABC transporter (11,13–15), and by a low-affinity transporter, AraE, which binds to the inner membrane and makes use of electrochemical potential to intake the arabinose (11,16,17). This system exhibits wide variability in the timing of activation and in the rates of accumulation of inducer molecules (18). It has been hypothesized that this is due to the cell-to-cell variability in the numbers of proteins responsible for the intake of arabinose (18). Interestingly, if the intake gene *araE* is placed under the control of a constitutive promoter the intake rates become more homogenous (19–21), suggesting that the diversity in the number of intake proteins is a non-negligible source of cell-to-cell variability in the kinetics of the arabinose utilization system (12).

Evidence suggests that when the intracellular concentration of arabinose exceeds a threshold, the dimeric AraC protein activates the genes that code for the proteins responsible for the intake (AraE and AraFGH) and for the catabolism of arabinose (*araBAD*) (11,22). In the absence of arabinose, AraC binds two half-sites on the DNA (I_1_ and O_2_) and promotes the formation of a DNA loop that prevents access of RNA polymerases to the promoters in that region (P*_C_* and P*_BAD_*). When bound by arabinose, AraC binds instead to the adjacent I_1_ and I_2_ half-sites. The resulting configuration promotes transcription initiation at P*_BAD_* (11).

Transcription initiation is a complex, multi-stepped process (23,24). *In vitro* measurements suggest that this process has at least two to three rate limiting steps (25,26). It starts when the RNA polymerase binds to the promoter region of the DNA molecule, forming the closed complex, which is followed by the open complex formation and promoter escape (27,28). The RNA polymerase then elongates the nascent RNA (28). Evidence suggests that, in general, initiation is much longer in duration than elongation (26,29). Recent *in vivo* measurements of the kinetics of initiation of P*_lac/ara-__1_* and P*_tetA_* promoters have shown that RNA production under the control of these promoters is a sub-Poissonian process (8–10). These studies also support the existence of multiple steps at the stage of initiation, significantly limiting the rate of RNA production, as suggested by *in vitro* measurements (30).

Here, we investigate the degree of contribution of the process of intake of arabinose and of the process of transcription under the control of P_BAD_ to the cell-to-cell diversity in RNA production. Namely, we report measurements of the *in vivo* kinetics of induction and transcript production of P*_BAD_* with single-molecule sensitivity, making use of the MS2d-GFP tagging of RNA in *E. coli* (31). For that, in each cell, we measure the waiting time until the first RNA is produced after induction and the subsequent intervals between consecutive transcript productions. For comparison, we conduct the same measurements for P*_lac/ara-__1_* when induced by either of its two inducers, arabinose and IPTG.

## MATERIALS AND METHODS

### Strains and plasmids

*Escherichia coli* strain DH5α-PRO was generously provided by I. Golding, University of Illinois and contains the construct PROTET-K133, carrying P*_L__tetO-__1_*-MS2d-GFP (31), along with a new construct, pMK-BAC (P*_BAD_*-mRFP1-MS2-96bs), which is a single-copy F-based vector carrying a sequence coding for a monomeric red fluorescent protein (mRFP1) followed by a 96 binding site array under the control of P*_BAD_* (cloning information provided in Supplementary Methods) (see also Supplementary Figures S1 and S2). The strain with plasmids P*_L__tetO-__1_*-MS2d-GFP and pIG-BAC (P*_lac/ara-__1_*-mRFP1-MS2-96bs) (32) was used as well. The DH5α-PRO strain [identical to Z1 (31)] is a genuine producer of AraC (33). No modifications were made to the chromosome of this strain in our experiments.

### Media and growth conditions

Cells were grown overnight at 30°C with aeration and shaking in Luria-Bertani (LB) medium, supplemented with antibiotics according to the plasmids. The cells were diluted in fresh M63 medium and allowed to grow until an optical density of OD_600_ ≈0.3–0.5. To attain full induction of the MS2d-GFP reporter, cells were pre-incubated for 40 min with 100 ng/ml anhydrotetracycline (aTc, IBA GmbH). The same protocol was used for each strain.

### Microscopy

For microscopy measurements, cells were pelleted and re-suspended in ∼50 µl of fresh M63 medium. Afterwards, few microlitres of cells were placed between a 3% agarose gel pad made with medium and a glass coverslip before assembling the imaging chamber (FCS2, Bioptechs). Before the starting of the experiment, the chamber was heated to 37°C.

Cells were visualized in a Nikon Eclipse (TE2000-U, Nikon, Japan) inverted microscope with C1 confocal laser-scanning system using a ×100 Apo TIRF objective. A flow of fresh, pre-warmed M63 medium containing the inducer was provided with a peristaltic pump at a rate of 1 ml/min. Images were taken once per minute for 2 h, and the laser shutter was open only during the exposure time to minimize photobleaching. The peristaltic pump was initialized at the same time as the collection of the time series. For image acquisition, we used Nikon EZ-C1 software. GFP fluorescence was measured using a 488 nm argon ion laser (Melles-Griot), 515/30 nm emission filter and a pixel dwell time of 3.36 µs (total image acquisition time of 3.5 s).

An interacting multiple model filter-based autofocus strategy (34) was used to correct focus drift in time series acquisitions. The method estimates the focal drift using an interacting multiple model filter algorithm to predict the focal drift at time *t* based on the measurement at *t*-1. It allows reducing the number of required images at different z-planes for drift correction, thus minimizing photobleaching.

### Data and image analysis

Data and images were analysed using custom software written in MATLAB 2011b (MathWorks). Cells were detected from fluorescence images by a semi-automatic method described previously (8). In time series, the area occupied by each cell was manually masked. Principal component analysis was used to obtain the dimensions and orientation of the cells within each mask. Fluorescent spots in the cells were automatically segmented using density estimation with a Gaussian kernel (35) and Otsu’s thresholding (36). Finally, background-corrected spot intensities were calculated and summed to produce the total spot intensity in each cell.

**Figure 1.**
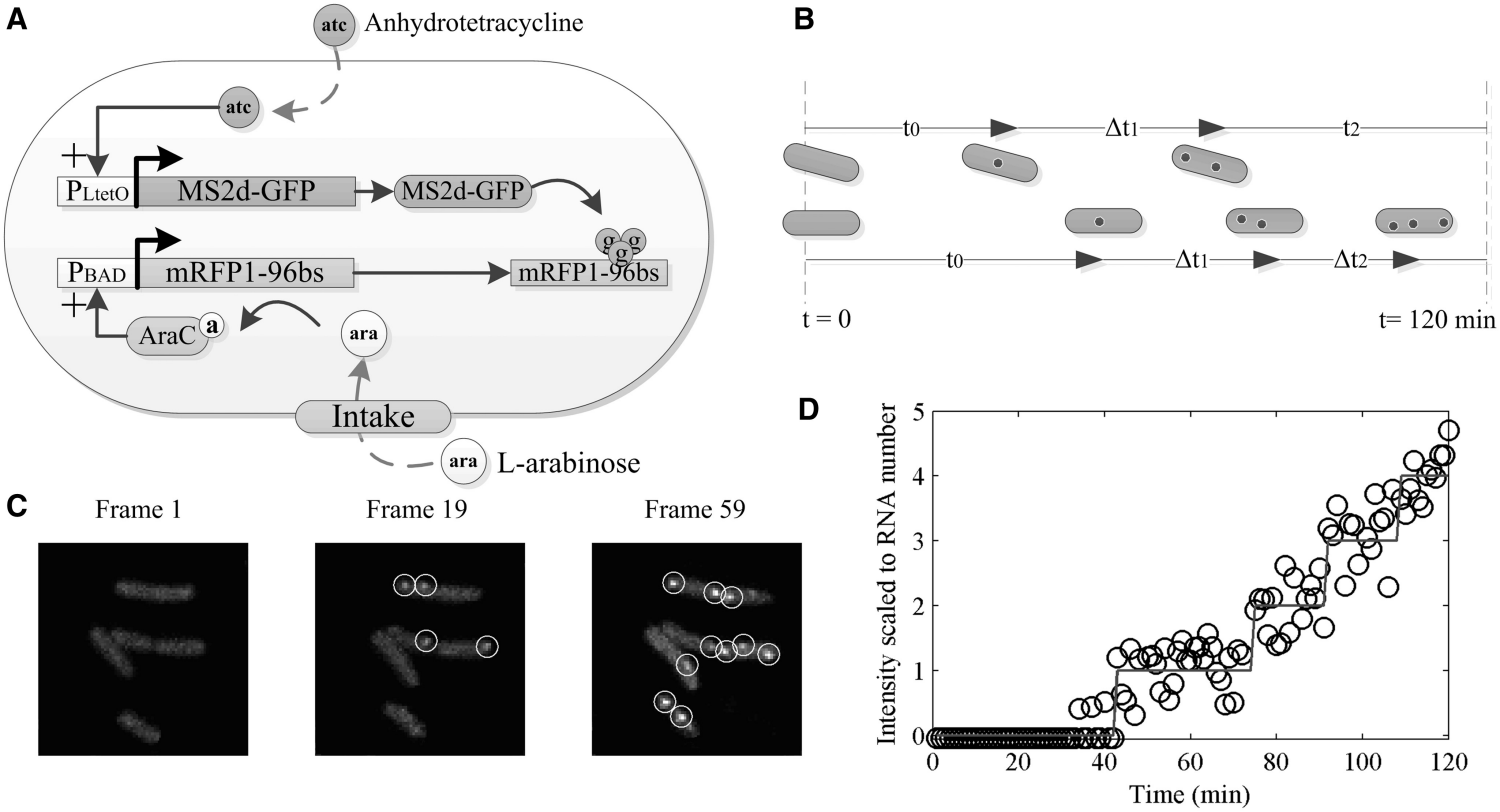
Measurement system. (**A**) Components of the detection system. The expression of the tagging protein, MS2d-GFP, is controlled by P*_LtetO_* (33) and is inducible by anhydrotetracycline (aTc). The target RNA contains an mRFP1 coding region, followed by an array of 96 MS2d-binding sites. Expression of the target RNA is controlled by P*_BAD_* whose activity is regulated by AraC and the inducer l-arabinose. The target construct is on a single-copy F-plasmid. The tagging construct is on a medium-copy vector. (**B**) Figurative description of the waiting time for the first RNA production (t_0_) and intervals between subsequent productions (Δt). Images are taken once per minute for 2 h. (**C**) Example of *E. coli* cells expressing MS2d-GFP and target RNA. GFP-tagged RNA molecules are marked by circles. (**D**) Time course of total intensity of spots in a cell (circles) and monotone piecewise-constant fit (line).

Moments of appearance of novel target RNA molecules in each cell were obtained from time-lapse fluorescence images by fitting the corrected total spots intensity over time in each cell to a monotone piecewise-constant function by least squares (37). The number of terms was selected using the F-test with a *P*-value of 0.01. Each jump corresponds to the production of a single RNA molecule (37). An example of this procedure is shown in Figure 1D. For more details on the image analysis see (8). Note that, in cells that do not contain target RNA molecules at the start of the measurements, the number of novel RNA molecules detected since the start of the measurements until a given moment equals the total number of RNA molecules in the cell at that moment.

Because some cells already contained target RNA molecules at the start of the measurement, the total RNA numbers within cells at a given moment in time is obtained using a different method. Specifically, when comparing measurements using MS2d-GFP tagging and using plate reader (Supplementary Figure S4), the total number of MS2d-GFP–tagged RNA molecules was extracted from the total spot intensity distribution, obtained from all cells in an image obtained at a given moment after induction. For this, the first peak of the obtained distribution is set to correspond to the intensity of a single-RNA molecule. The number of tagged RNAs in each spot can be estimated by dividing its intensity by that of the first peak (32).

## RESULTS

### Experimental design

To study the kinetics of expression of P*_BAD_*, we detect individual RNA molecules, as these are produced in live cells and register when these events occur. For this, we placed the P_BAD_ promoter on a single-copy F-plasmid, followed by a coding region for mRFP1 and an array of 96 binding sites for MS2d-GFP–tagging proteins (32) (Figure 1A). The expression of MS2d-GFP is controlled by P*_T__etO_*, which is activated before the gene of interest so that sufficient MS2d-GFP proteins are present when target RNA molecules appear. Induction of P*_BAD_* and image acquisitions is initialized simultaneously (Figure 1B). For this, we use a temperature-controlled imaging chamber and a peristaltic pump for introducing inducers and fresh media. From the fluorescence images, using semi-automated cell segmentation and tracking (Figure 1C) (8), we measure in each cell the time for the first RNA to appear (named ‘waiting time’, t_0_), as well as the subsequent intervals between consecutive RNA productions, Δt, until cell division occurs or until the end of the measurement period (Figure 1D).

Given that values of t_0_ can only be obtained from cells of the first generation (i.e. cells already on the slide when the measurement begins), and as cells that do not divide in the first 2 h will not, in general, divide afterwards, we limited the measurement period to 2 h for simplicity. This was possible, as this period also proved to be sufficient to acquire enough samples of Δt.

From cells born during the measurement period, we only extract intervals between consecutive RNA productions, not waiting times, as these contain inducers by inheritance. We detected no difference in the distributions of intervals obtained from such cells and cells already present when induction is initiated. Finally, we observed ∼0.2 RNA molecules per cell, at the moment preceding induction, because of spurious transcription events. Cells where a target RNA was already present at the start of the measurement were also not used to obtain values of t_0_.

First, we compared by quantitative polymerase chain reaction the RNA production from the F-plasmid and from the native gene under the control of P*_BAD_* (Supplementary Methods). Using 16S rRNA as reference gene, we observe similar trend in activity over time in the native promoter and in the one on the F-plasmid (Supplementary Figure S3).

We next compare expression levels of the target gene, when assessed by independent methods, for two induction levels, namely, 0.1 and 1% l-arabinose (Supplementary Methods). In Supplementary Figure S4A and B, we show the temporal variation after induction in mean numbers of MS2d-GFP–tagged RNA molecules in cell populations and in the fluorescence intensity of RFP measured by plate reader, respectively.

The plate reader measurements of mRFP1 levels, 2 h after induction in liquid culture, show a fold change of 1.67 times when l-arabinose is increased from 0.1 to 1%. The MS2d-GFP *in vivo* detection method shows a fold change of 1.74 between these same conditions, showing that the results from the two methods are in accordance. From this and the previous experiment, we also conclude that the MS2d-GFP tagging method accurately detects RNA production of the target gene, and that the target gene behaves similarly to the natural system.

We also assessed for what range of inducer concentrations is the target gene under full induction. We measured with the plate reader its expression for varying inducer concentration, 2 h after induction. From Supplementary Figure S5, maximum induction is achieved for 1% arabinose. Here onwards, unless stated otherwise, we use this concentration to assess the kinetics of RNA production under the control of P*_BAD_*.

### First RNA and intervals between consecutive RNA molecules in individual cells

From the time-lapse images acquired with confocal microscopy, after induction, we measure in each cell both t_0_ and subsequent values of Δt. t_0_ is expected to include the time for arabinose to enter the cell via the intake mechanism, the time to find the promoter and release the repressor and also the time for the recruitment of the RNA polymerase and subsequent production of the first target RNA. The latter process includes events such as the closed and the open complex formation at the promoter region, as well as the elongation time. Both the elongation time and the time for MS2d-GFP to bind to a target RNA are expected to be negligible in comparison with the duration of the intake and of transcription initiation (8,12,31). Meanwhile, Δt should depend only on the events in transcription initiation (37).

**Figure 2.**
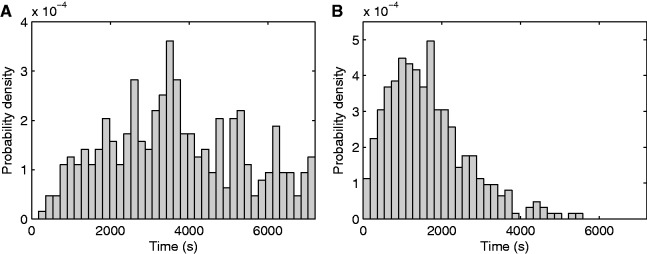
Kinetics of the intake and production. (**A**) Probability density distribution of waiting times (*µ* = 3071 s, σ = 1711 s) for the first RNA to be produced in cells induced by 1% l-arabinose (354 data points). (**B**) Probability density distribution of intervals between transcription events for P*_BAD_* when induced by 1% l-arabinose (*µ* = 1672 s, σ = 1012 s) (347 data points).

The distribution of values of the waiting times, t_0_, is shown in Figure 2A. Cells were induced in the gel with fresh media and 1% arabinose. The distribution is broad, as the waiting times spread through the measurement time and has a mean of 3071 s.

The distribution of intervals between consecutive productions of target RNA molecules (Δt) is shown in Figure 2B. This production is a sub-Poissonian process, as the normalized variance (σ^2^/µ^2^) of the distribution is 0.37. Similar conclusions were obtained from measurements of the *in vivo* kinetics of RNA production under the control of P*_lac/ara-__1_* and P*_tetA_* (9,10).

The distributions in Figure 2A and B differ significantly. We verified this with a statistical testing of equality of two empirical distributions, the Kolmogorov–Smirnov (K–S) test. We obtained a *P*-value of 2.8 × 10^−^^18^, much smaller than 0.05, which allows rejecting the null hypothesis of similarity. We conclude that in the case of P*_BAD_* and the arabinose intake mechanism, the time of intake of inducers affects significantly both mean and standard deviation of RNA numbers in individual cells, long after induction. Finally, note that the difference between the distributions of t_0_ and Δt provides evidence that the activity of P*_BAD_* changes significantly with induction. Otherwise, these two distributions should not differ significantly, as they would both result, e.g. from spurious transcription events alone.

One recent study (12) also focuses on the *in vivo* induction kinetics of P*_BAD_*. This study uses measurements of GFP levels in cell populations, whose expression is controlled by P*_BAD_* (inserted into a medium-copy vector) and a model to extrapolate the mean activation time of the promoter, after induction. Assuming a threshold for GFP levels to consider the promoter as active, the mean appearance time of GFP after induction was ∼960 s. By considering several features of the measurement system, including the mean maturation time of GFP, a value was then extrapolated for the expected activation time of the promoter, namely, ∼250 s. This does not include the time for transcription to be completed, once the closed complex is formed. This study thus predicts a faster mean initiation time than what our direct measurements indicate (∼3000 s). Two main reasons exist for this difference. First, in the mutant used previously (12), the chromosomal *araBAD* operon is deleted, avoiding the negative feedback mechanism, which likely fastens the response time significantly. Additionally, gene expression was assessed from a medium-copy vector, which should respond much faster than the single-copy vector system used here, as its response time depends on the fastest of the response times of several promoter copies. Thus, we find that the results reported previously (12) and ours are in agreement. For example, while observing mean waiting times one order of magnitude longer, we do observe RNA molecules appearing in some of the cells within a time scale of 200–400 s after induction. Therefore, provided the usage of a multi-copy vector instead of the single-copy vector used here, we expect mean waiting times one order of magnitude smaller and thus in agreement with the measurements described previously (12).

### Correlations between consecutive processes

To study whether the durations of the processes of intake and of transcription initiation are correlated, we first assessed whether consecutive intervals of Δt in individual cells are correlated. We measured the Pearson correlation from 101 pairs of consecutive intervals, and found it to be 0.16. We obtained a *P*-value of 0.11, assuming no correlation as the null hypothesis, which implies that we cannot prove that the correlation is significant. This is in agreement with previous studies of P*_lac/ara-__1_* kinetics, which also indicate inexistence of correlation between durations of consecutive intervals between RNA productions (8).

We next assessed whether the distributions of t_0_ and values of Δt (Figure 2A and B) are correlated. Note that t_0_ and the Δt are of similar order of magnitude as the measurement period. This introduces artificial correlations between t_0_ and Δt of individual cells, as, e.g. a cell with a large t_0_ is expected to exhibit smaller than average Δt values, as larger intervals would not be detected during the measurement period as likely as in cells with smaller values of t_0_. To remove these artificial correlations between t_0_ and Δt of individual cells, in this assessment, we only considered RNA productions for a certain window size (Supplementary Methods and Supplementary Figure S6). This window is set so as to maximize the number of data points that can be extracted from the measurements.

From the windowed data, we calculated the Pearson correlation between t_0_ and Δt values in individual cells to be −0.15. We calculated a *P*-value of 0.18 assuming no correlation as the null hypothesis, which implies that we cannot prove that the correlation is significant. This result is in line with (12), which reports a lack of correlation between initiation of protein expression and subsequent rate of protein synthesis in individual cells.

### Dynamics of induction and of transcription initiation under different induction schemes

The distinctiveness of the distributions of t_0_ and Δt of P*_BAD_*, as assessed by the K–S test, suggests that they are, partially, the result of different processes. Although t_0_ ought to depend on the kinetics of intake of arabinose and on the first transcription initiation event, Δt values ought to depend mostly on the kinetics of transcription initiation events alone.

These assumptions arise from the following. First, *in vitro* and *in vivo* measurements (26,38) suggest that transcription initiation (including closed and open complex formation) is a long-duration, multi-step process, usually taking 10^2^–10^3 ^s in bacterial promoters (10,25,26,37,38). Other events that need to occur before the appearance of a target RNA because of the tagging of the MS2d-GFP are not expected to affect Δt significantly. These are transcription elongation and the tagging by multiple MS2d-GFP. Elongation of the target RNA was measured to take only tens of seconds (31). Also, the tagging occurs at a rate that makes the RNA visible during elongation or shortly after (31).

To test the two assumptions, we measured the distributions of t_0_ and Δt for another promoter, P*_lac/ara-__1_*, in two conditions. P*_lac/ara-__1_* can be induced either by IPTG or by arabinose (as P*_BAD_*), or by both inducers simultaneously (9). According to our assumption, the distribution of t_0_ of P*_BAD_* is expected to be similar to that of P*_lac/ara-__1_* when the latter is induced by arabinose, because of depending on the same intake mechanism, whereas it should differ significantly when P*_lac/ara-__1_* is induced by IPTG, given the different intake mechanisms of IPTG.

We measured the distributions of t_0_ and Δt for P*_lac/ara-__1_* when induced by IPTG alone and when induced by arabinose alone (Table 1). We used the same concentration of arabinose as when inducing P*_BAD_*. The IPTG concentration used is the one required for maximum induction of P*_lac/ara-__1_* (33). Results in Table 1 follow the windowing procedure described earlier in the text. The table shows mean, standard deviation and square of the coefficient of variation (µ^2^/σ^2^) of t_0_ and of Δt for the two promoters, each of which in two induction schemes.

**Table 1.** Measurements of t_0_ and Δt

Promoter	Inducer	No. of samples (Δt)	µ_Δt_ (s)	σ_Δt_ (s)	σ^2^/µ^2^	No. samples (t_0_)	µ_t0_ (s)	σ_t0_ (s)	σ^2^/µ^2^
P*_BAD_*	1% arabinose	102	1440.6	532.8	0.14	84	2885.0	1159.8	0.16
P*_BAD_*	0.1% arabinose	78	1475.4	481.2	0.11	70	3519.4	1236.2	0.12
P*_lac/ara-__1_*	1% arabinose	149	1516.5	516.0	0.12	125	2832.5	1184.6	0.17
P*_lac/ara-__1_*	1 mM IPTG	485	1314.4	576.0	0.19	286	2697.0	913.6	0.11

The table shows the mean (µ), the standard deviation (σ) and the normalized variance (σ^2^/µ^2^) of the measured distributions of t_0_ and Δt.

We first assessed the distinctiveness of the distributions of t_0_ and Δt by the K–S test, for each promoter in each condition (Table 2). In all cases, these two distributions differ in a statistical sense. This is in agreement with the assumption that although both Δt and t_0_ depend on the kinetics of initiation at the promoter, only t_0_ depends on the kinetics of intake of inducers.

**Table 2.** *P*-values of the Kolmogorov–Smirnov test between t_0_ and Δt distributions for each promoter and induction condition

Promoter	Inducer	*P*-value
P*_BAD_*	1% arabinose	2.83 × 10^−18^
P*_BAD_*	0.1% arabinose	4.06 × 10^−21^
P*_lac/ara-__1_*	1% arabinose	2.48 × 10^−26^
P*_lac/ara-__1_*	1 mM IPTG	3.32 × 10^−72^

For *P* < 0.05, it is generally accepted that the hypothesis that the two distributions are the same should be rejected.

We next performed statistical tests to assess the distinctiveness between the induction kinetics (t_0_) of the two promoters (Table 3), when subject to the same inducer and when subject to different inducers. Also, we compared the effects of a different inducer concentration in the case of P*_BAD_*. From Table 3, when P*_BAD_* and P*_lac/ara-__1_* are induced with 1% arabinose, they exhibit distributions of t_0_ that cannot be distinguished. However, when P*_lac/ara-__1_* is induced with IPTG, the resulting t_0_ distribution is statistically distinguishable from that of P*_BAD_*, when induced by either 0.1 or 1% arabinose. It is also distinct from its own t_0_ distribution when induced by 1% arabinose. This statistically significant difference supports the hypothesis that the distributions of t_0_ are dependent on the kinetics of the intake system of the inducers, and that these differ for IPTG and arabinose.

**Table 3.** *P*-values of the Kolmogorov–Smirnov test between t_0_ distributions for each promoter and induction condition

	P*_BAD_* 1% arab	P*_BAD_* 0.1% arab	P*_lac/ara-__1_* 1% arab	P*_lac/ara-__1_* IPTG
P*_BAD_* 1% arab	1			
P*_BAD_* 0.1% arab	5.93 × 10^−4^	1		
P*_lac/ara-__1_* 1% arab	0.8533	1.10 × 10^−4^	1	
P*_lac/ara-__1_* IPTG	0.0126	4.49 × 10^–12^	0.0049	1

For *P* < 0.05, it is generally accepted that the hypothesis that the two distributions are the same should be rejected.

Finally, we observed that the distributions of t_0_ of P*_BAD_*, when induced by 0.1% and by 1% arabinose, are distinct. This is expected as the time for inducers to ‘first reach the promoter’ ought to depend on the inducer’s concentration.

### Kinetics of the intake process

The intake time of an inducer, here named ‘t_diff_’, differs from t_0_ in that it does not include the time for the first transcription initiation event to occur. Because of this, t_diff_ cannot be measured directly with the MS2-GFP–tagging method. We thus estimate the mean and variance of the distribution of values of t_diff_ by subtracting the means and variances of the Δt distribution from the t_0_ distribution. This method is based on the fact that we were unable to establish the existence of a correlation between the values of t_0_ and Δt. Given this, and as they are, at most, weakly correlated (Pearson correlation of −0.15), we assume that they are independent so as to be able to estimate the standard deviation of the duration of the intake process alone (note that the mean of this quantity can be estimated as described later in the text, regardless of the existence of dependence).

The estimated mean and a standard deviation of t_diff_ are similar for P*_BAD_* and for P*_lac/ara-__1_*, when induced with 1% arabinose. Namely, in both cases, we obtained a mean of ∼1400 s and a standard deviation of ∼1100 s. This is expected, given the usage of the same intake mechanism and inducer concentration. Importantly, when P*_lac/ara-__1_* is induced by IPTG, the standard deviation of t_diff_ is much smaller (∼700 s), whereas the mean is similar to when induced by arabinose (∼1400 s). This suggests that the intake of arabinose is a noisier process (concerning the uncertainty of the intake time) than the intake of IPTG. Finally, we find that in the case of P*_BAD_*, the concentration of arabinose affects the mean of t_diff_ significantly, as it equals ∼2000 s for 0.1% arabinose.

### Effect of the intake process on the temporal cell-to-cell diversity in RNA numbers

Because of being stochastic and thus variable in duration from one event to the next (i.e. it differs from one cell to the next), the intake process impacts on the diversity in RNA numbers of a cell population. This impact should decrease with time, after induction. We estimated the time during which the effect is tangible for each measurement condition. For this, we assume that values of t_0_ depend mostly on the intake of arabinose and on the first transcription initiation event at the start site of P*_BAD_*. Meanwhile, the distribution of intervals between consecutive RNAs is assumed to depend solely on the kinetics of transcription initiation (8,10,37).

The events determining Δt as well as t_0_ are modelled as *d*-step processes, each step with an exponentially distributed duration (Supplementary Methods) (37). From this assumption, it is possible, for a given number of steps, to find the duration of each step that best fits the measurements. We assume transcription initiation to be a three-step process, namely, the closed complex formation, the open complex formation and promoter escape (27,38), as evidence suggests that these are the most rate-limiting steps in normal conditions, i.e. the ones most contributing to the intervals between production of consecutive RNA molecules (26). This assumption also relies on recent studies (37) that indicate that assuming this number of steps suffices to generate distributions that cannot be distinguished, in a statistical sense, from measurements with accuracy and quantity of data similar to the measurements reported here. Finally, we assume the intake to be a two-step process, namely, the binding of extracellular arabinose to an uptake protein and, once bound, its translocation to the cytoplasm (12). The combination of the two processes (intake followed by transcription initiation) is, consequently, assumed to be a five-step process.

Assuming these numbers of steps and stable conditions (e.g. induction level), we searched for models that fit the distributions accurately enough so that the K–S test does not find differences between model and measurements. The *P*-values of these tests are shown in Supplementary Table S1 and show that in all but one case, it is possible to find a model that cannot be distinguished from the empirical distribution, in a statistical sense.

The case for which we could not find a model that fits the measurements is that of P*_BAD_* at 0.1% arabinose induction. This may be due to lack of sufficient data or because the model is unsuitable. Future studies are required to assert this. One explanation may be that, in this case, the distribution of intake times results from two distinct kinetics, one being the productions under induction and the other being spurious productions by promoters in the ‘non-induced’ state.

**Figure 3.**
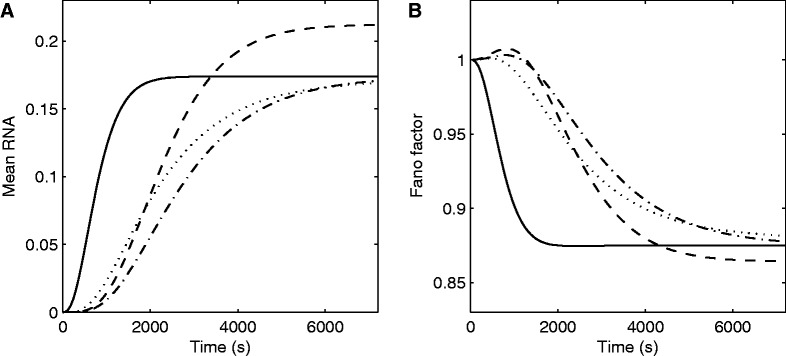
Mean and Fano factor of transient times for different models of intake and subsequent RNA production kinetics. Mean (**A**) and Fano factor (**B**) of RNA numbers as obtained by CME models of activation and expression. The models shown are that of P*_lac/ara-1_* with 1 mM IPTG (dashed line), P*_lac/ara-1_* with 1% arabinose (dotted line), P*_BAD_* with 1% arabinose (dash-dotted line) and P*_BAD_* with 1% arabinose and infinitely fast intake (solid line).

Given the models aforementioned and provided a rate of RNA degradation, it is possible to estimate the time it takes for the mean RNA numbers of a model cell population to reach equilibrium, as this time depends solely on the rate of degradation of RNAs and t_0_. We do not have measurements of the degradation rate of the target RNA, as the tagging with MS2d-GFP ‘immortalizes’ it for the duration of the measurements (32). Instead, the models in Figure 3 assume an RNA degradation rate of 5 min^−^^1^, which is within realistic intervals for *E. coli* (1).

From all of the aforementioned data, we estimated the mean times for RNA numbers to reach near-equilibrium, as well as the Fano factor of this quantity since the start of the simulations. Results are shown in Figure 3, as estimated for each of the models. Also shown is an estimation that assumes the model of transcription initiation of P*_BAD_* when induced by 1% arabinose, coupled with an infinitely fast intake.

In all cases, reaching equilibrium in mean RNA numbers takes >1 h, except when assuming infinitely fast intake, in which case the time to reach equilibrium is <0.5 h. Thus, for a time length as long as 1–2 h, the intake process has a non-negligible contribution on the mean and the on the cell-to-cell diversity in RNA numbers of the cell populations. From Figure 3A, one also observes different shapes in the curves of P*_lac/ara-__1_* when induced by IPTG (dashed line) and when induced by arabinose (dotted line), because of differing intake kinetics.

From Figure 3B, the contribution of the intake kinetics on the cell-to-cell variability in RNA numbers is also significant. For example, the kinetics of intake causes an increase in the Fano factor in the initial moments not observable in the case of infinitely fast intake.

We also tested models of P*_BAD_* induced by 1% arabinose (normal and infinitely fast intake) with other RNA degradation rates (Supplementary Figure S7), within realistic intervals (1). Aside from assessing the degree of dependency on the intake time and degradation rate, one also observes from the figure that although the latter determines the rate at which the system reaches equilibrium, the former acts as a delay towards reaching the numbers at equilibrium. Further, one can see that the intake step adds diversity to the RNA numbers in the cells, during the transient to reach equilibrium.

## DISCUSSION

We measured, at the single-cell level, how long it takes for the first RNA under the control of P*_BAD_* to be produced, followed the introduction of the inducer in the media. Also, we measured the subsequent intervals between consecutive RNA productions. From the intervals between transcription events, we determined that RNA production under the control of P*_BAD_* is a sub-Poissonian process. Two recent studies reached similar conclusions for P*_lac/ara-__1_* and P*_tetA_*, for all induction conditions tested (9,10). We hypothesize that this may be a common phenomenon because of the kinetic properties of the process of transcription initiation in bacteria, in particular, because of its multi-stepped nature.

From the distributions of the time, it takes for the appearance of the first RNA in each cell when under the control of P*_BAD_* and of P*_lac/ara-__1_*, for different induction conditions, we assessed the effect of the kinetics of the intake process on the mean and cell-to-cell diversity in RNA numbers of cell populations. Relevantly, this effect was found to be tangible for a long period after induction. Also, we verified that different intake mechanisms differ significantly not only in mean but also in the degree of variability of the intake time, and that this has a non-negligible effect on RNA population statistics.

Given the aforementioned data, and considering that natural environments are fluctuating, we expect the kinetics of cellular intake mechanisms to have a significant effect on the degree of phenotypic diversity of cell populations. Finally, we expect the methodology used here to assess the *in vivo* kinetics of intake of arabinose and of IPTG to be applicable to any gene of interest. Such studies should provide valuable insight into the adaptability of prokaryotic organisms to environmental changes and stress. They should also provide a better understanding of the observed cell-to-cell phenotypic diversity in *E. coli* when in fluctuating environments.

## SUPPLEMENTARY DATA

Supplementary Data are available at NAR Online: Supplementary Table 1, Supplementary Figures 1–7, Supplementary Methods and Supplementary References [39–43].

## FUNDING

The Academy of Finland [257603 to A.S.R.]; the Finnish Funding Agency for Technology and Innovation [40226/12 to O.Y.-H.]; the Foundation for Science and Technology, Portugal [PTDC/BBB-MET/1084/2012 to A.S.R.]. Funding for open access charge: Academy of Finland [257603, 2012 to A.S.R.].

*Conflict of interest statement.* The funders had no role in study design, data collection and analysis, decision to publish, or preparation of the manuscript.

## Supplementary Material

Supplementary Data
