# Supplementary material for: In vivo single-molecule kinetics of activation and subsequent activity of the arabinose promoter
Source: Nucleic Acids Res. 2013 May 3;41(13):6544–52. doi: 10.1093/nar/gkt350 (PMC3711423; doi:10.1093/nar/gkt350)
Supplement: Supplementary Data [file supp_41_13_6544__index.html]

In vivo single-molecule kinetics of activation and subsequent activity of the arabinose promoter — In vivo single-molecule kinetics of activation and subsequent activity of the arabinose promoter — Supplementary Data 

# *In vivo* single-molecule kinetics of activation and subsequent activity of the arabinose promoter

## Supplementary Data

files

**Files in this Data Supplement:**

- Supplementary Data - pdf file
