# Supplementary material for: In vivo single-molecule kinetics of activation and subsequent activity of the arabinose promoter
Source: Nucleic Acids Res. 2013 May 3;41(13):6544–52. doi: 10.1093/nar/gkt350 (PMC3711423; doi:10.1093/nar/gkt350)
Supplement: Supplementary Data [file supp_gkt350_nar-00839-v-2013-File002.pdf]

Supplementary Information

In vivo single-molecule kinetics of activation and subsequent activity of the arabinose promoter

Jarno Mäkelä[1], Meenakshisundaram Kandhavelu[1], Samuel M.D. Oliveira[1], Jerome G. Chandraseelan[1], Jason Lloyd-Price[1], Juha Peltonen[1], Olli Yli-Harja[1,2] and Andre S. Ribeiro[1,*]

[1] Laboratory of Biosystem Dynamics, Department of Signal Processing, Tampere University of Technology, P.O. Box 553, 33101 Tampere, Finland.
[2] Institute for Systems Biology, 1441N 34th St, Seattle, WA, 98103-8904, USA

* Corresponding author: andre.ribeiro@tut.fi (Andre S. Ribeiro)

Supplementary Methods

Chemicals

Bacterial cell cultures were grown in two media, namely Luria-Bertani (LB) and M63. The chemical components of LB broth (Tryptone, Yeast extract and NaCl) were purchased from LabM (UK). For M63 media, the following components were used: 2 mM $MgSO_4.7H_20$ (Sigma-Aldrich, USA), 7.6 mM $(NH_4)_2SO_4$ (Sigma Life Science, USA), 30µM $FeSO_4.7H_2O$ (Sigma Life Science, USA), 1 mM EDTA (Sigma Life Science, USA), 60 mM KH_2PO_4 (Sigma Life Science, USA), Glycerol 0.5 % (Sigma Life Science, USA), and Casaminoacids 0.1 % (Fluka Analytical, USA). Isopropyl β-$_D$-1-thiogalactopyranoside (IPTG), $_L$-(+)-Arabinose and anhydrotetracycline (aTc) used for induction of the cells and the antibiotics (100 mg/ml kanamycin and 35 mg/ml chloramphenicol) were purchased from Sigma-Aldrich (USA). Agarose (Sigma Life Science, USA) was used for the microscopic slide gel preparation.

Bacterial Strain

Cloning and expression experiments were performed in *E. coli* DH5α-PRO strain (Clontech; identical to DH5α-Z1 (31). The strain information is: deoR, endA1, gyrA96, hsdR17($r_{k-}m_{k+}$), recA1, relA1, supE44, thi-1, Δ(lacZYA-argF)U169, Φ80δlacZΔM15, F-, λ-, P_{N25}/tetR, P_{lacIq}/lacI, and Sp^R. Frag1A: F-, rha-, thi, gal, $lacZ_{am}$, ΔacrAB::kan^R, P_{N25}/tetR, P_{lacIq}/lacI, and Sp^R. Frag1B: F-, rha-, thi, gal, $lacZ_{am}$, P_{N25}/tetR, P_{laciq}/lacI, and Sp^R. The P_{N25}/tetR, P_{laciq}/lacI, Sp^R cassette was transferred from *DH5αPRO* to Frag1 to generate Frag1B by P1 transduction. The ΔacrAB:kan^R cassette was transferred from KZM120 to Frag1B, so as to generate Frag1A.

Construction of the pMK-BAC vector

To construct the pMK-BAC (P_{BAD}-mRFP1-96 binding site (96 BS) array, the following plasmids were used: a plasmid with mRFP1 plus 96bs array region in the BAC vector, originally designed and generously provided by Prof. Ido Golding ($P_{lac/ara-1}$- mRFP1-96 bs) (32). To amplify the construct containing the AraC and pBAD promoter region from the pGLO vector (Biorad), a primer set was designed as follows:

Ara_AatII-Fw-5´CCTAAGACGTCATCGATGCATAATGTGCC 3´
Ara_AatII-Rv-5´CCTTGATGACGTCATGTATATCTCCTTCTTAAAGTTA3´

The target BAD promoter region along with AraC coding region from the pGLO vector was amplified and inserted into the pIG-BAC vector by standard molecular biology techniques. The construct was verified by sequencing with the appropriate primers and transformed into the *E. coli* DH5α-PRO strain carrying the bacterial expression vector pPROTET.E (Clontech) coding for MS2d–GFP. For more details see Supplementary Figures 1 and 2.

Plate reader experiment

The mean fluorescence of RFP under the control of P_{BAD} was measured with a microplate fluorometer (Fluoroskan Ascent, Thermo Scientific). 200 ml of cells at OD600 ≈ 0.5 were induced with 0.1 % or 1 % L-arabinose and placed on 96 well microplate. From this, cells were measured for 2 hours for relative fluorescence levels of mRFP1 protein (excitation and emission wavelengths were 584 nm and 607 nm, respectively). The cell density was kept identical in all wells of the plate for all conditions.

Quantitative PCR for mean mRNA quantification

The change in the rate of transcription of genes araB and mRFP was studied using qPCR. *E. coli* DH5α-PRO cells containing the constructs were grown as described in the section describing the microscopy measurements. Cells were grown overnight at 30°C with aeration, diluted into fresh medium and allowed to grow at the appropriate temperature of the experiment until an optical density of OD_{600} ≈ 0.3-0.5 was reached. For the experiment, 5 ml of cells were pre-incubated with 100 ng/ml of aTc to induce the expression of MS2d-GFP. 1 % $_{L}$-arabinose was used for induction of the BAD promoter, 30 minutes after induction, the first sample was taken. From then onwards, samples were taken at an interval of 60 minutes. Rifampicin was added to the samples immediately, so as to prevent further transcription and the cells were fixed with RNA protect reagent immediately followed by enzymatic lysis using Tris-EDTA lysozyme buffer (pH 8.3). RNA was purified from each sample by RNeasy mini-kit (Qiagen). The total RNA was separated by electrophoresis through a 1 % agarose gel and stained with SYBR Safe DNA Gel Stain. The RNA was found intact with discreet bands for 16 S and 23 S ribosomal RNAs. To ensure purity of the RNA samples, they were subject to treatment with DNase free of RNase, to remove residual DNA. The yield of RNA obtained was 0.4 – 0.6 mg/ml. Approximately 40 ng of RNA was used for cDNA synthesis using iSCRIPT reverse transcription super mix (Biorad) according to the manufacturer’s instructions.

Quantification of cDNA was performed by real-time PCR using SYBR-green supermix with primers for the amplification of target and reference genes at a concentration of 200nM. Primers specific to AraB (Forward: 5' GGTACTTCCACCTGCGACAT 3', Reverse: 5' CAACCTGACCGCAAATACCT 3') and mRFP genes (Forward: 5' TACGAC GCCGAGGTCAAG 3' and Reverse: 5' TTGTGGGAGGTGATGTCCA 3') were designed using PRIMER3 (39), the length of the amplicon for the target and reference were maintained at 90bp. The sequence of the primers for the reference gene 16S rRNA (EcoCyc Accession Number: EG30090) (Forward: 5' CGTCAGCTCGTGTTGTGAA 3' and Reverse: 5' GGACCGCTGGCAACAAAG 3') and the primers were obtained from Thermo Scientific. The level of 16s rRNA was used to normalize the expression data of each target gene. 10 ng of cDNA was used as a template. The cycling protocol used was 94 °C for 15 s, 51 °C for 30 s, and 72 °C for 30 s, up to 39 cycles. The amplification was monitored in real time by measuring the fluorescence intensities at the end of each cycle. The experiment was performed in triplicates along with the No-RT and no template controls. The volume used for each reaction was 25 µl in low-profile tube strips in a MiniOpticon Real time PCR system (Biorad). The Cq values were obtained from the CFX ManagerTM Software and the fold change of expression of the target gene was analysed by normalizing against the reference gene according to the Livak method (40). See Supplementary Figure 3 for the results.

Normalization between samples of the distributions of time intervals

The observation time for the production of RNAs is two hours. In some cells, the intervals between transcription events (Δt) are of this order of magnitude. This causes shorter intervals to be 'favored'. This is more likely to occur in cells where the waiting time for the first RNA to be produced (t_0) is longer, since the remaining observation time is shorter. This introduces an artificial anti-correlation between t0 and Δt in individual cells. Similar correlations are introduced by different division times as well, i.e., shorter division times hamper the collection of longer Δt samples.

Thus, prior to determine if any real correlation exists between t_0 and Δt in individual cells, it is necessary to remove these artificial sources of anti-correlation due to the limits in the measurement period. For this, in all cells, all intervals between consecutive RNAs were collected only for a time window of size t_c after the previous production. The value of t_c is identical in all cells. This causes the probability of appearance of the next RNA molecule during that period to be uniform for all cells, if the underlying process is in fact identical in all cells.

This restriction in the collection of values of Δt is made when assessing correlations between t_0 and Δt and when comparing these two distributions between conditions. When imposing the restriction, we thus consider only cells that produce at least 2 RNA molecules during their life time and measurement period. The value of t_c was selected so as to maximize the number of data points collectable from the data sets. Here, t_c was set to 39 minutes (see Supplementary Figure S6).

Fitting the empirical distributions to a sum of *d*-exponential variates

The arabinose intake mechanism can be described by a single Michaelis-Menten function (41). Since the backward reaction of the intake process is slower than the forward reaction (12), the intake process is modeled, roughly, by a sequence of non-reversible reactions. Interestingly, we found from the measurements and the inference procedure, evidence of two steps at this stage (exponential in duration), which is in agreement with the number of forward steps assumed in other studies for this process (12). Finally, transcription initiation, which follows the intake process, can also be modeled by a 3-step exponential model according recent in vivo measurements (9, 10). Thus, we fit the measured distributions of t_0 to a 5-step exponential model.

To fit the empirical distribution with a sum of *d*-exponential variates (of possibly unequal rates), we select the exponential rate parameters $\lambda_1, \dots, \lambda_d$ such that the Kolmogorov-Smirnov (K-S) statistic is minimized. That is, parameters are selected as $\hat{\theta} = \arg\max_{\theta=\lambda_1,\dots,\lambda_d} \sup_x |F_\theta(x) - G(x)|$, where $F_\theta(x)$ is the cumulative distribution function (CDF) of a sum of d exponentials with parameters $\theta = (\lambda_1, \dots, \lambda_d)$, and $G(x)$ is the CDF of the empirical distribution.

$$F_{\theta=L_1,\dots,L_d}(X) := \sum_{i=1}^{d} ((1 - e^{-L_i x}) \prod_{\substack{j=1 \\ j\neq i}}^{d} \frac{L_j}{L_j - L_i})$$

The parameter values θ are found using a nonlinear numerical optimizer. This method is convenient, since if the K-S test was rejected for the parameters $\hat{\theta}$, such a test would also be rejected for any other set of parameters θ in this family of fitted distributions, indicating that these distributions are inappropriate models of the data. The results of the fitting are shown in Table S1.

As a final note, the model assumed above can be considered as the simplest possible, i.e., each step is an elementary reaction of the form $A \xrightarrow{c} B$, with a constant probability of occurring per unit time. This entails that the distributions of intervals between steps are exponential (42). Notably, the inferred distributions and the experimental data are statistically indistinguishable by the K-S test, which implies that there is no evidence to assume that the model is wrong (see Table S1).

CME solution

To estimate the effect of the intake on the cell-to-cell diversity in RNA numbers we made use of direct integration of the Chemical Master Equation (CME) of the model described in the previous section, using the Finite State Projection algorithm (43). This method truncates the infinite state space of the CME such that the amount of probability outside the truncated region is negligible. In all cases, we truncated the state space at 20 RNA molecules. This number sufficed for this space to contain virtually all of the total probability in the system. The probability mass vector at each time moment is then solved by numerically integrating the truncated CME. From this distribution over time, we calculate mean, variance, and Fano factor of RNA molecules of a model at each moment.

Supplementary Figures

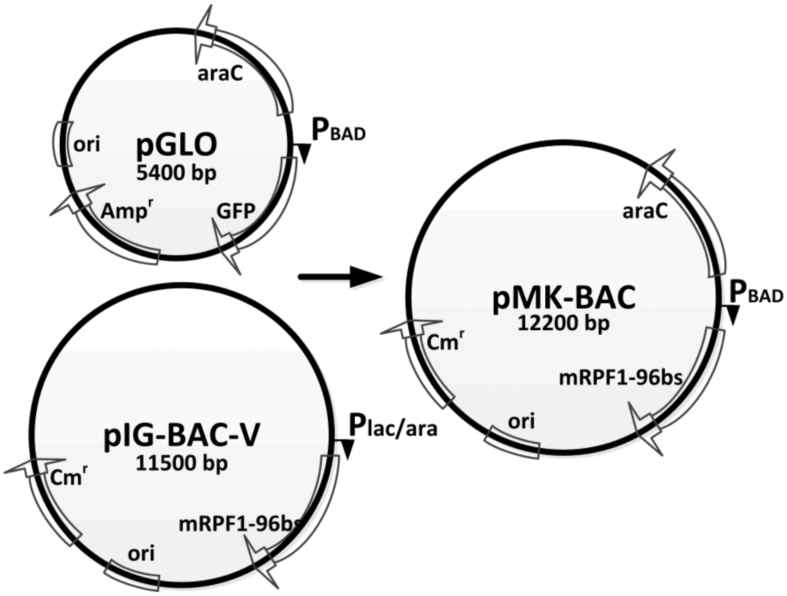

Figure S1. Plasmids used for the pMK-BAC construction. The pMK-BAC(P_{BAD}-mRFP1-96bs) plasmid was engineered by linking the amplified region, containing the P_{BAD} promoter and the araC gene, obtained from pGLO, to the pIG-BAC expression vector, without the lac/ara-1 promoter, obtained from pIG-BAC($P_{lac/ara-1}$- mRFP1-96 bs)-V.

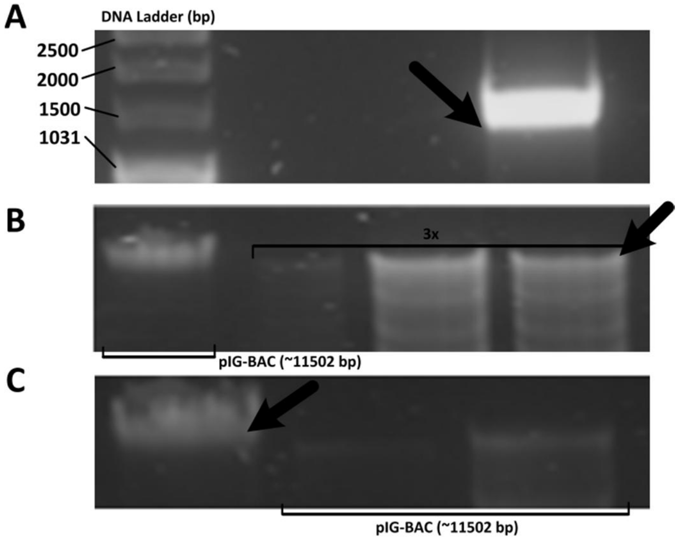

Figure S2. Split gels of the plasmid construction. (A) The PCR fragment of 1347bp amplified from pGLO with the appropriate primers. (B) Lanes containing pIG-BAC-V without the $P_{lac/ara-1}$ promoter region (10849bp), and the pIG-BAC-V expression plasmid (11502bp. (C) The pMK-BAC plasmid (12196bp) containing the araC-P_{BAD} amplified fragment inserted to the BAC expression vector, and the pIG-BAC-V (11502bp). Note the black arrows indicating the bands.

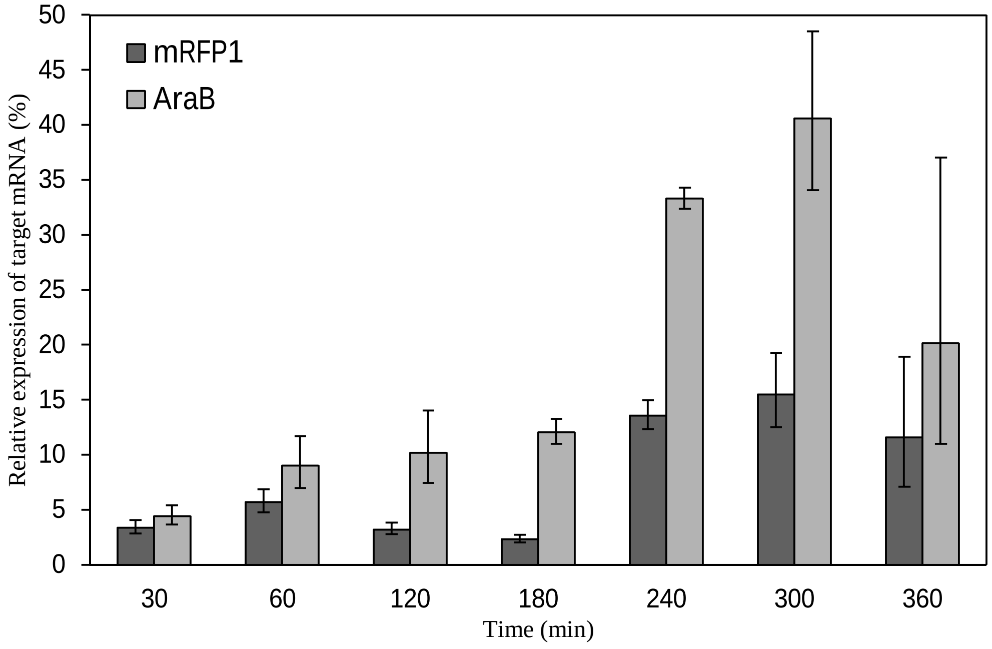

Figure S3. Q-PCR of the native and of the target gene. Q-PCR of RNA expression of the native, integrated AraB gene and of the mRFP1 probe in the F-plasmid, as a function of time, when subject to induction by 1% L-arabinose in liquid culture. The standard deviation bars are from three independent experiments.

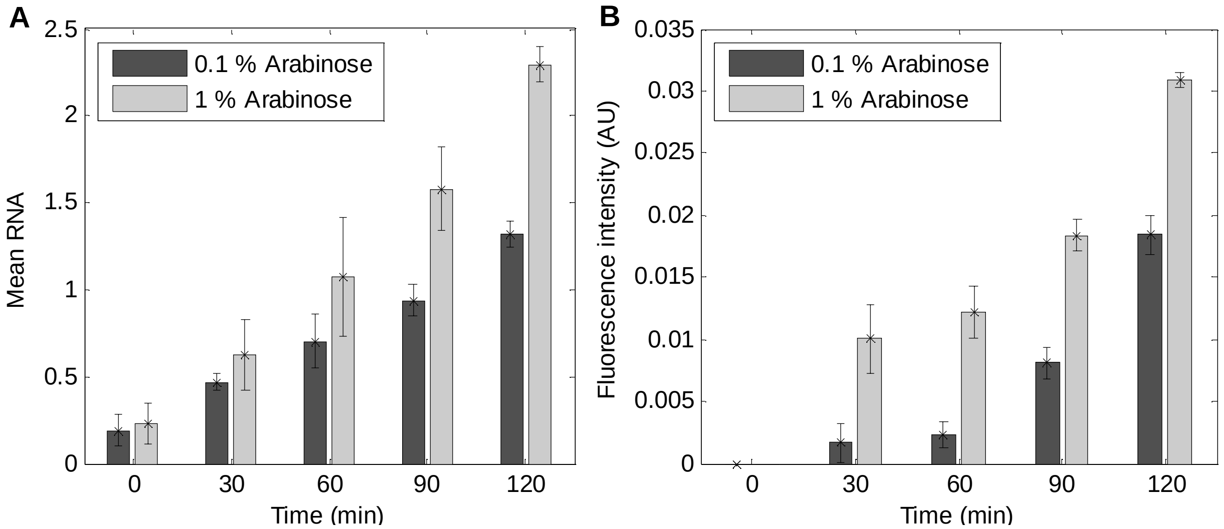

Figure S4. MS2-GFP measurement of RNA numbers compared with Plate reader results. (A) RNA numbers over time measured in vivo with the MS2-GFP method for 0.1 % and 1 % L-arabinose. Mean and standard deviation of RNA numbers in individual cells were calculated for each sample separately. Error bars show the standard error of the mean from independent measurements (3 measurements) (B) Fluorescent intensity of RFP over time for 0.1 % and 1 % L-arabinose as measured by Plate reader. Error bars show the standard error of the mean obtained from 8 wells.

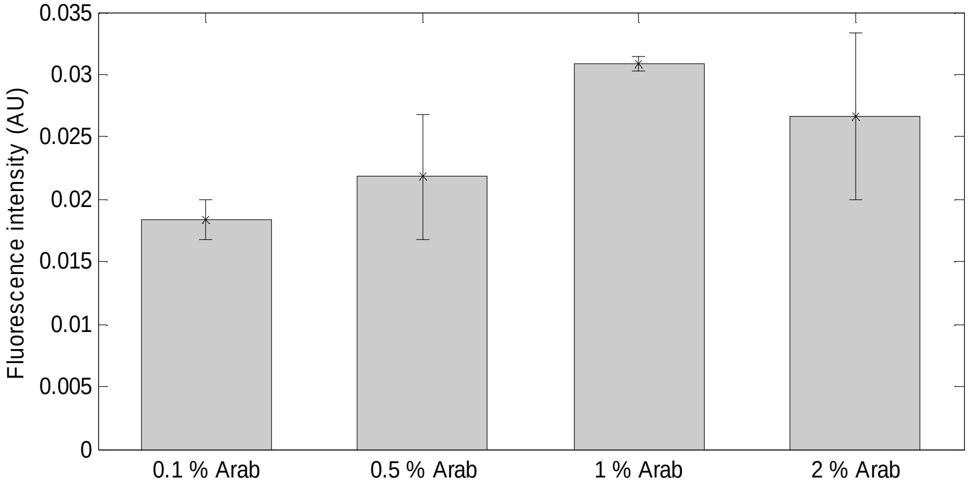

Figure S5. Gene expression as measured by Plate Reader. Comparison of different inducer concentrations by plate reader measurements, 2 hours following induction. Maximum induction is achieved with 1 % L-arabinose. Error bars show the standard error of the mean obtained from 8 wells.

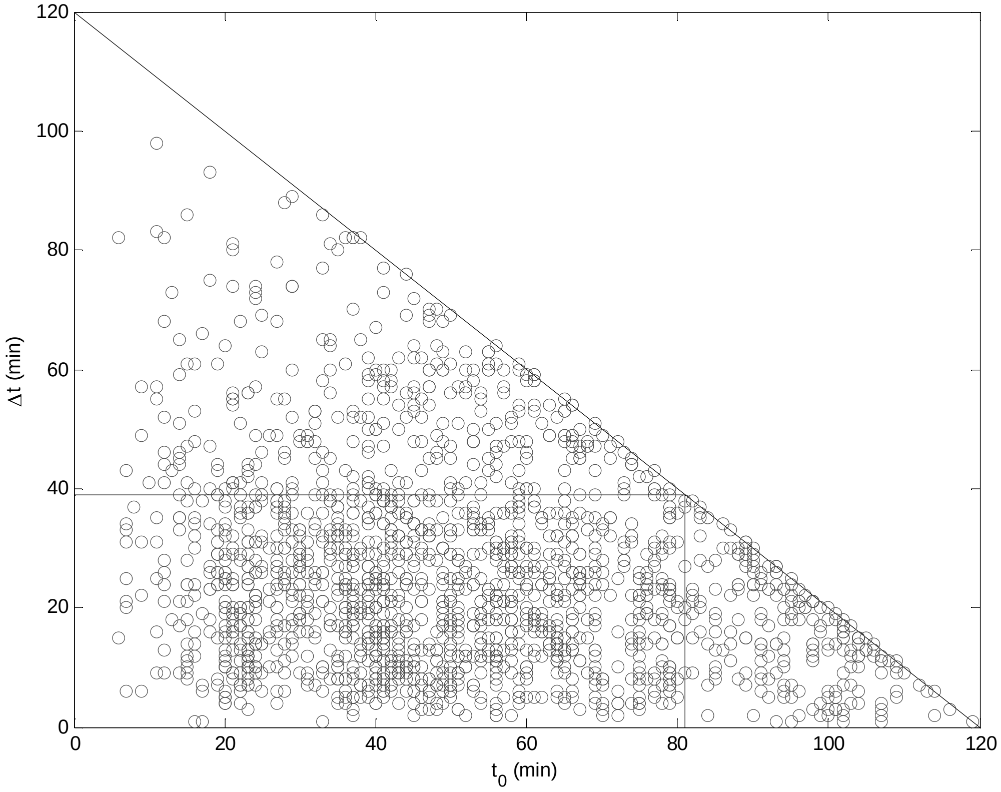

Figure S6. Normalization of the data. The values of t_0 and the corresponding values of the first Δt in each cell. The diagonal line is the total observation time (120 min). Vertical and horizontal (t_c = 39 min) lines define the intervals that meet the requirements for un-biasedness.

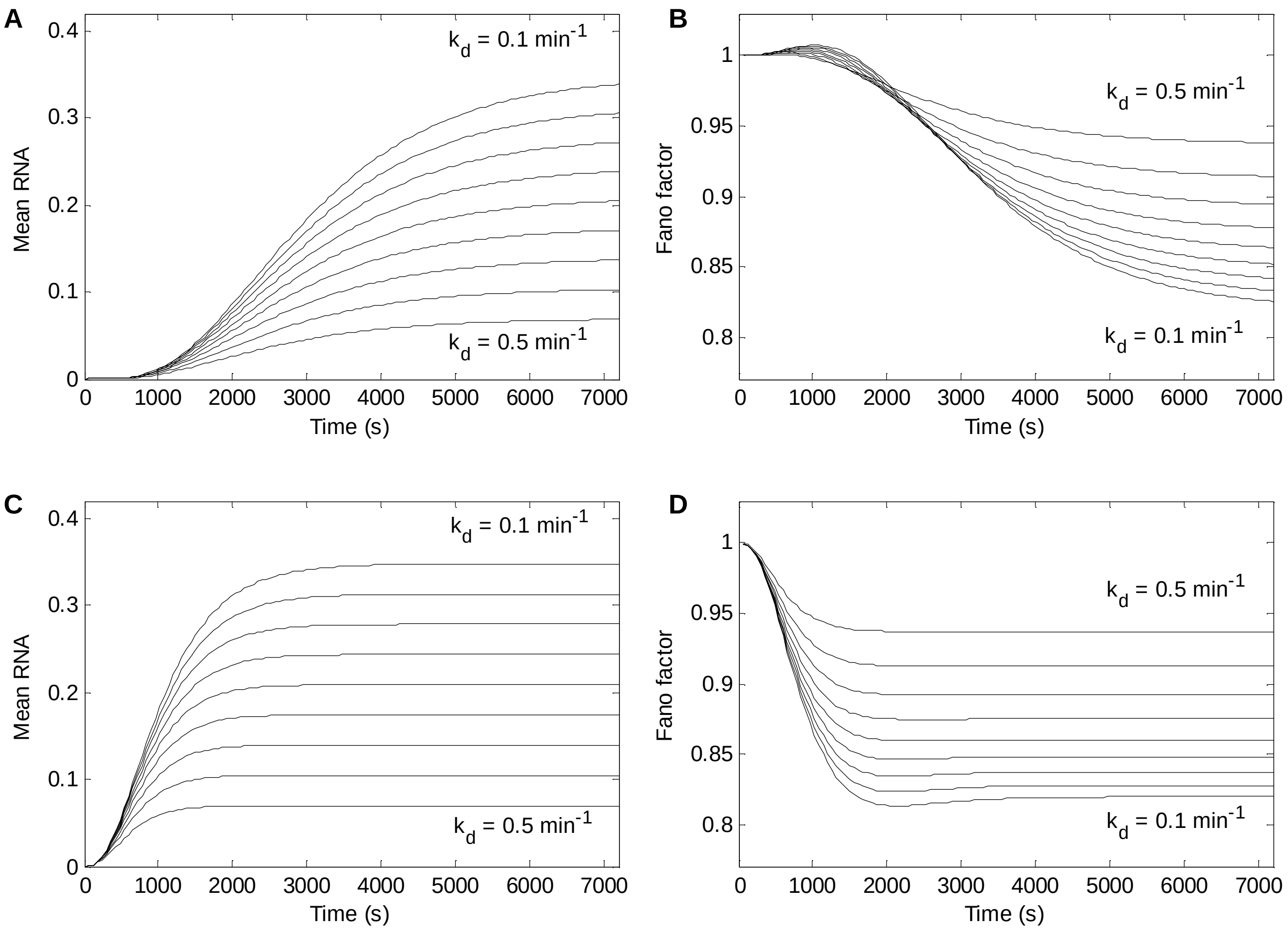

Figure S7. Models with different degradation rates. The degradation rate was set to the following values: 0.1 min^{-1}, 0.111 min^{-1}, 0.125 min^{-1}, 0.143 min^{-1}, 0.167 min^{-1}, 0.2 min^{-1}, 0.25 min^{-1}, 0.333 min^{-1}, 0.5 min^{-1}. In the figures, only the highest and the lowest values are marked. Mean RNA numbers shown for (A) P_{BAD} with 1 % Arabinose and for (C) P_{BAD} with 1 % Arabinose and infinitely fast intake. Fano factors of RNA numbers are shown for (B) P_{BAD} with 1 % Arabinose and, (D) P_{BAD} with 1 % Arabinose and infinitely fast intake.

Supplementary Table

	p-value for t_0	p-value for Δt
P_{BAD} 1 % arabinose	0.2613	0.8930
P_{BAD} 0.1 % arabinose	0.0020	0.5728
$P_{lac/ara-1}$ 1 % arabinose	0.1759	0.3826
$P_{lac/ara-1}$ 1 mM IPTG	0.1155	0.2413

Table S1. Results of the K-S fitting. Asymptotic p-values of the Kolmogorov-Smirnov goodness-of-fit test when fitting the empirical distribution with a sum of 5-exponential variates in the case of t_0 and of 3-exponential variates in the case of Δt. We compare these p-values with a standard value of 0.05.
